# High-flow oxygen via tracheostomy facilitates weaning from prolonged mechanical ventilation in patients with restrictive pulmonary dysfunction: two case reports

**DOI:** 10.1186/s13256-018-1832-7

**Published:** 2018-10-12

**Authors:** Chieko Mitaka, Masahiko Odoh, Daizoh Satoh, Tadasuke Hashiguchi, Eiichi Inada

**Affiliations:** 1 Department of Anesthesiology and Pain Medicine, Juntendo University, 2-1-1, Hongo, Bunkyo-ku, Tokyo, 113-8421 Japan; 2 Department of Esophageal and Gastroenterological Surgery, Juntendo University, Tokyo, Japan

**Keywords:** High-flow oxygen, Hypercapnia, Restrictive pulmonary dysfunction, Tracheostomy, Weaning

## Abstract

**Background:**

Weaning from prolonged mechanical ventilation is extremely difficult in tracheostomized patients with restrictive pulmonary dysfunction. High-flow oxygen via tracheostomy supplies heated and humidified oxygen gas at > 10 L/minute. However, little has been reported on the use of high-flow oxygen via tracheostomy during weaning from ventilators in patients with restrictive pulmonary dysfunction. We report successful weaning from ventilators in patients with restrictive pulmonary dysfunction using high-flow oxygen via tracheostomy.

**Case presentation:**

The first patient is a 78-year-old Japanese man with severe pneumococcal pneumonia who was mechanically ventilated for more than 1 month after esophagectomy for esophageal cancer. After he underwent tracheostomy because of prolonged mechanical ventilation, restrictive pulmonary dysfunction appeared: tidal volume 230–240 mL and static compliance 14–15 mL/cmH_2_O with 10 cmH_2_O pressure support ventilation. He was weaned from the ventilator under inspiratory support with high-flow oxygen via tracheostomy over a period of 16 days (flow at 40 L/minute and fraction of inspired oxygen of 0.25). The second patient is a 69-year-old Japanese man who developed aspiration pneumonia after esophagectomy and received prolonged mechanical ventilation via tracheostomy. He developed restrictive pulmonary dysfunction. High-flow oxygen via tracheostomy (flow at 40 L/minute with fraction of inspired oxygen of 0.25) was administered with measurement of the airway pressure and at the entrance of the tracheostomy tube. The measured values were as follows: 0.21–0.3 cmH_2_O, 0.21–0.56 cmH_2_O, 0.54–0.91 cmH_2_O, 0.76–2.01 cmH_2_O, 1.17–2.01 cmH_2_O, and 1.76–2.01 cmH_2_O at 10 L/minute, 20 L/minute, 30 L/minute, 40 L/minute, 50 L/minute, and 60 L/minute, respectively. The airway pressures were continuously positive and did not become negative even during inspiration, suggesting that high-flow oxygen via tracheostomy reduces inspiratory effort. He was weaned from the ventilator under inspiratory support with high-flow oxygen via tracheostomy over a period of 12 days.

**Conclusions:**

High-flow oxygen via tracheostomy may reduce the inspiratory effort and enhance tidal volume by delivering high-flow oxygen and facilitate weaning from prolonged mechanical ventilation in patients with restrictive pulmonary dysfunction.

## Background

Weaning from prolonged mechanical ventilation is extremely difficult in tracheostomized patients with restrictive pulmonary dysfunction. Mechanical ventilation for longer than 21 days is associated with increased mortality, health care utilization, and health care costs in critically ill patients [1]. High-flow oxygen via tracheostomy (HFT) delivered by Optiflow™ (OPT 870; Fisher & Paykel Healthcare Ltd, Auckland, New Zealand) is a method to supply heated and humidified oxygen gas at > 10 L/minute. Although high-flow oxygen through nasal cannula (HFNC) has demonstrated clinical benefits, such as improved oxygenation [2] and reduced 90-day mortality [3], the mechanisms of HFT may differ from those of HFNC. Furthermore, minimal information has been reported on the use of HFT during weaning from ventilators in patients with restrictive pulmonary dysfunction. Our hypothesis is that HFT increases tidal volume by reducing inspiratory effort in patients with restrictive pulmonary dysfunction. Here, we report two cases in which respiratory support with HFT proved useful for weaning from prolonged mechanical ventilation in patients with restrictive pulmonary dysfunction.

## Case presentation

### Case 1

A 78-year-old (height 163.3 cm, weight 61.3 kg) Japanese man was admitted to our intensive care unit (ICU) after esophagectomy with gastric reconstruction for esophageal cancer. His body temperature was 36.9 °C and heart rate was 96 beats/minute. His blood pressure was 148/68 mmHg on ICU admission. Physical and neurological examinations were not significant. His past medical history included appendectomy (8 years of age) and lumber canal stenosis (70 years of age). He did not smoke cigarettes but drank Japanese alcohol (360 ml/day). Regarding family history, his elder brother died of esophageal cancer.

**Fig. 1:**
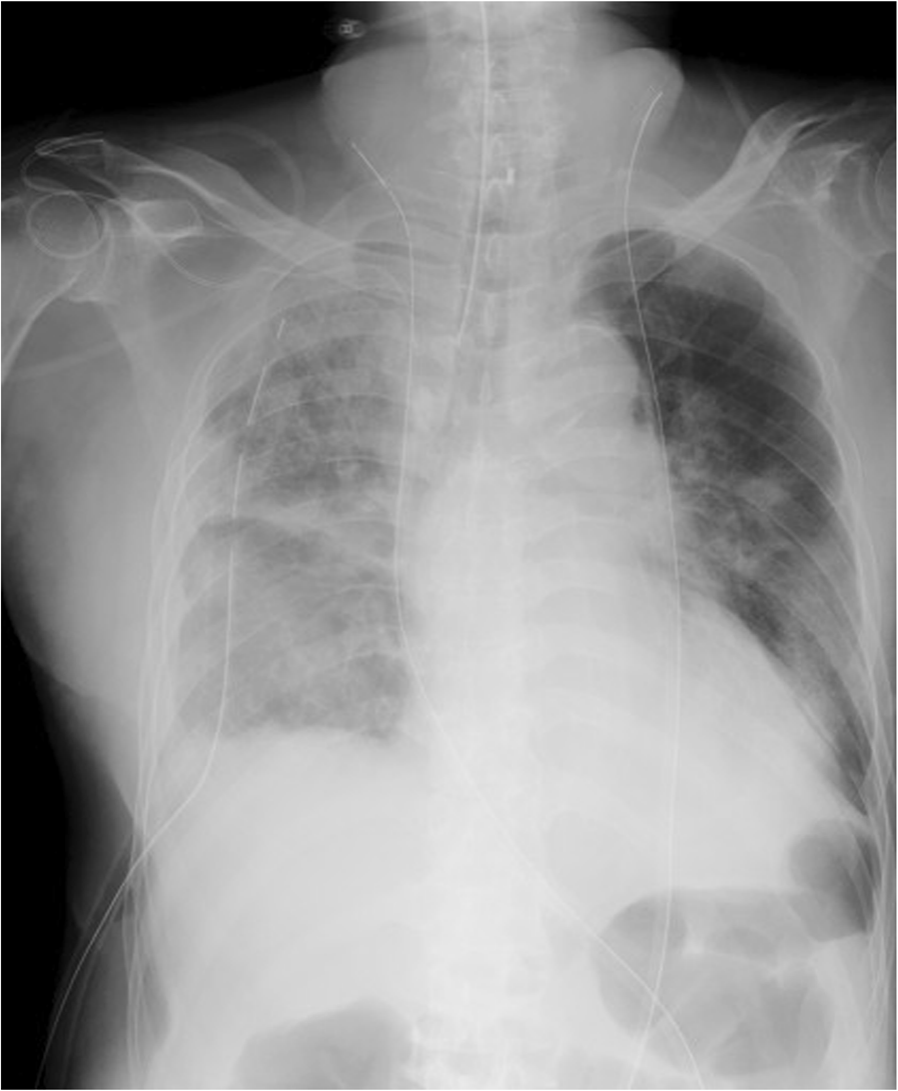
Chest X-ray depicting diffuse bilateral pulmonary infiltrates predominantly in the right lung with pleural effusion after tracheal intubation on intensive care unit day 5

On ICU day 5, he developed new onset fever up to 38.0 °C, increase in sputum, and hypoxemia with 90% arterial oxygen saturation by pulse oximetry (SpO_2_) with mask oxygen at 10 L/minute with bilateral pulmonary coarse crackles. Therefore, he was intubated. A chest X-ray revealed diffuse bilateral pulmonary infiltrates predominantly in his right lung with pleural effusion (Fig. 1). Laboratory findings revealed white blood cell count 5.7 × 10^9^/L, red blood cell count 2.25 × 10^12^/L, hemoglobin 7.3 g/dL, hematocrit 21.4%, platelet 145 × 10^9^/L, aspartate aminotransferase 54 U/L, alanine aminotransferase 55 U/L, total bilirubin 4.11 mg/dL, albumin 2.2 g/dL, urea nitrogen 38 mg/dL, creatinine 0.83 mg/dL, C-reactive protein 18.7 mg/dL, and urinary creatinine 95 mg/dL after intubation. Because sputum culture revealed *Streptococcus pneumoniae* on ICU day 7, the antibiotic was changed from cefmetazole to meropenem.

He underwent a long period of mechanical ventilation, ultimately undergoing tracheostomy on ICU day 38. Although his oxygenation was good with partial pressure of arterial oxygen/fraction of inspired oxygen (PaO_2_/F_I_O_2_) > 300 mmHg, restrictive pulmonary dysfunction developed: tidal volume 230–240 mL, static compliance 14–15 mL/cmH_2_O with 10 cmH_2_O pressure support ventilation, respiratory rate 34 beats/minute, and partial pressure of arterial carbon dioxide (PaCO_2_) 46 mmHg. We tried to wean him from mechanical ventilation with support by HFT setting the flow at 40 L/minute with F_I_O_2_ of 0.25 because the maximum inspiratory flow of the ventilator was 40 L/minute during 10 cmH_2_O pressure support ventilation. By setting the flow at the same rate as the ventilator, we hoped to reduce his inspiratory effort. HFT was used in the daytime, and a ventilator with 5 cmH_2_O pressure support with 5 cmH_2_O positive end-expiratory pressure (PEEP) was used at night for the first 8 days. In the next 8 days, HFT was used around the clock. Table 1 presents the respiratory parameters during HFT. Under respiratory support with HFT, his condition was stable, and his physiotherapy rehabilitation continued uneventfully. The HFT optimally delivered humidified gas, which improved the thinning of his bronchial secretions. He was discharged from the ICU to the general ward on ICU day 127. His physiotherapy rehabilitation continued for 2 months, and he was transferred to a rehabilitation hospital on day 201 of hospitalization.

**Table 1:** Changes in respiratory parameters during high-flow oxygen via tracheostomy in Case 1

HFT day	1	2	3	4	5	6	7	8	9	10	11	12	13	14	15	16
HFT setting																
F_I_O_2_	0.25	0.25	0.25	0.25	0.25	0.25	0.25	0.25	0.25	0.25	0.25	0.25	0.25	0.25	0.25	0.25
Flow (L/minute)	40	40	40	40	40	40	40	20	15	15	15	15	15	15	15	15
RR (breaths/minute)	40	30	35	37	26	30	35	36	35	27	29	26	22	20	22	21
SpO_2_ (%)	97	98	97	97	96	98	97	98	96	96	96	96	98	95	98	96
etCO_2_ (mmHg)	47	41	40	39	42	42	40	41	39	35	34	35	37	35	38	41
V_T_ (mL)				240				260			300			300		
Arterial blood gases																
pH				7.46							7.47					
PaCO_2_ (mmHg)				47							44					
PaO_2_ (mmHg)				92							87					
HCO_3_^−^ (mmol/L)				32.5							31.2					

Abbreviations: *etCO*_*2*_ end-tidal carbon dioxide, *F*_*I*_*O*_*2*_ fraction of inspired oxygen, *HFT* high-flow oxygen via tracheostomy, *HCO*_*3*_^*−*^ bicarbonate ion, *PaCO*_*2*_ partial pressure of arterial carbon dioxide, *PaO*_*2*_ partial pressure of arterial oxygen, *RR* respiratory rate, *SpO*_*2*_ arterial oxygen saturation by pulse oximetry, *V*_*T*_ tidal volume (measured by Wright respirometer)

### Case 2

**Fig. 2:**
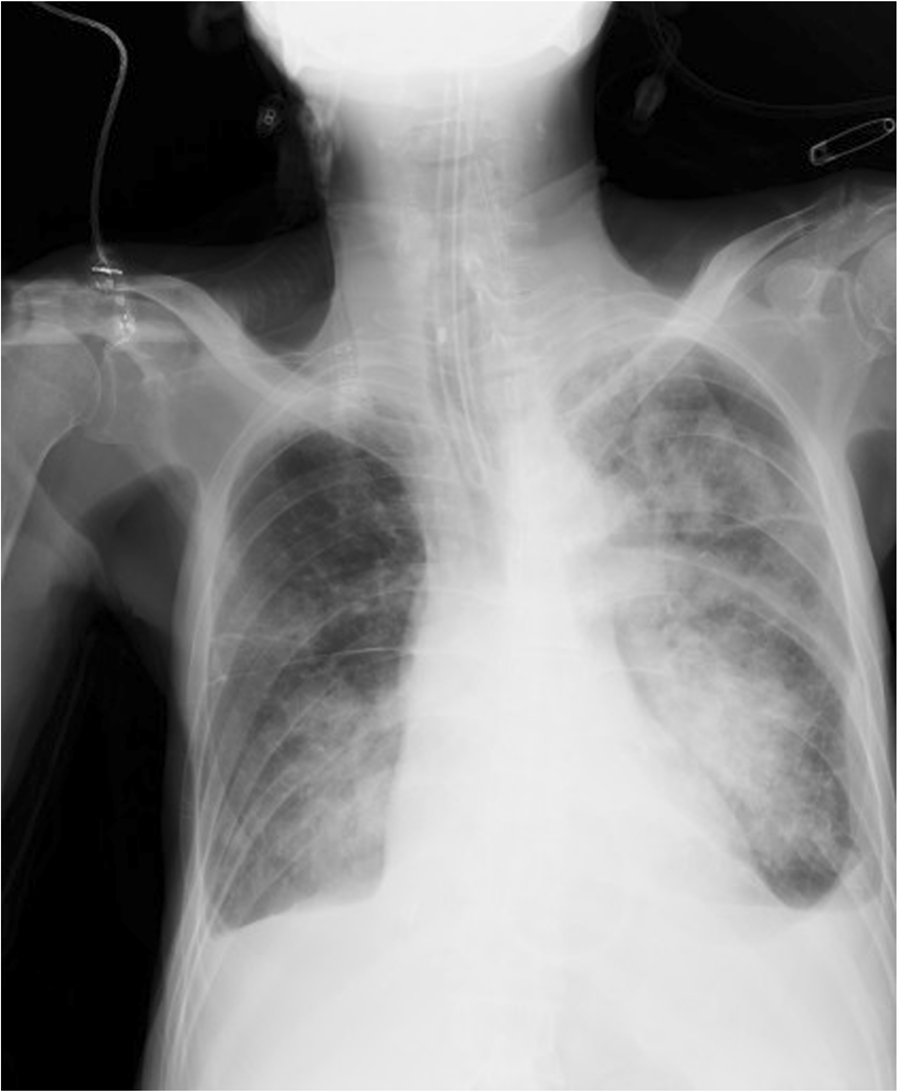
Chest X-ray depicting diffuse bilateral pulmonary infiltrates after tracheal intubation on intensive care unit day 1

A 69-year-old (height 160.0 cm, weight 37.1 kg) Japanese man was admitted to our ICU from the emergency room due to severe dyspnea. His past medical history included extracorporeal shock wave lithotripsy due to urinary calculus (56 years of age), endoscopic colon polypectomy because of colon polyps (66 years of age), and esophagectomy with gastric reconstruction for esophageal cancer after a stint of preoperative chemoradiation therapy (66 years of age). He smoked 30 cigarettes/day for 40 years and drank whisky (1 bottle/4 days). He was not on any medication. Regarding family history, his grandmother died of liver cancer. On physical examination, he was confused and restless, and his breathing was shallow with reduced air entry to both lungs. A neurological examination was not significant. His vital signs were as follows: heart rate 122 beats/minute, blood pressure 80/58 mmHg, respiratory rate 26 breaths/minute, and temperature 38.7 °C. Arterial blood gases exhibited respiratory acidosis: pH 7.21, PaCO_2_ 117 mmHg, PaO_2_ 76 mmHg, and bicarbonate ion (HCO_3_^−^) 45.9 mmol/L with mask oxygen at 6 L/minute. Even bag-valve-mask ventilation could not provide proper ventilation. He was immediately intubated, and numerous food particles, such as beans and rice, were aspirated from his trachea. Therefore, we removed these food particles by bronchoscope as soon as possible. A chest X-ray revealed diffuse bilateral pulmonary infiltrates (Fig. 2). He was diagnosed as having aspiration pneumonia and placed on mechanical ventilation. Laboratory findings revealed white blood cell count 0.9 × 10^9^/L, red blood cell count 4.66 × 10^12^/L, hemoglobin 12.1 g/dL, hematocrit 41.0%, platelet 297 × 10^9^/L, aspartate aminotransferase 17 U/L, alanine aminotransferase 7 U/L, total bilirubin 0.59 mg/dL, albumin 2.2 g/dL, urea nitrogen 26 mg/dL, creatinine 0.75 mg/dL, and C-reactive protein 1.7 mg/dL on ICU admission.

He underwent tracheostomy on ICU day 32 due to prolonged mechanical ventilation. Although his oxygenation was good (PaO_2_/F_I_O_2_ > 300 mmHg), he had restrictive pulmonary dysfunction: tidal volume 210–220 mL, static compliance 16–17 mL/cmH_2_O with 10 cmH_2_O pressure support ventilation, respiratory rate 30 beats/minute and PaCO_2_ 46 mmHg. We tried to wean him from mechanical ventilation with support by HFT (flow at 40 L/minute with F_I_O_2_ of 0.25). Table 2 presents the respiratory parameters over the 4-day period HFT was administered. On HFT day 4, his arterial blood gases were pH 7.41, PaCO_2_ 58 mmHg, PaO_2_ 68 mmHg, and HCO_3_^−^ 35.7 mmol/L. Judging his condition as satisfactory, we switched from HFT to a 3 L/minute oxygen T-piece. One hour after the T-piece was commenced, he complained of dyspnea and his arterial blood gases moderately worsened (pH 7.34, PaCO_2_ 72 mmHg, PaO_2_ 106 mmHg, and HCO_3_^−^ 37.3 mmol/L). At that time, his tidal volume was 200 mL. We decided to switch back to HFT (flow at 40 L/minute with F_I_O_2_ of 0.25), and his arterial blood gases improved 1 hour later: pH 7.40, PaCO_2_ 60 mmHg, PaO_2_ 71 mmHg, and HCO_3_^−^ 35.9 mmol/L. On HFT day 5, his PaCO_2_ increased to 70 mmHg. This condition suggested respiratory muscle fatigue; however, no complaints of dyspnea were noted. We decided to apply the HFT at daytime and switch to a ventilator (10 cmH_2_O pressure support with 5 cmH_2_O PEEP) at night, and the treatment regimen was continued accordingly for the next 8 days.

**Table 2:** Changes in respiratory parameters during high-flow oxygen via tracheostomy in Case 2

HFT day	1	2	3	4	5	6	7	8	9	10	11	12
HFT setting												
F_I_O_2_	0.3	0.25	0.25	0.25	0.3	0.3	0.3	0.3	0.3	0.3	0.3	0.3
Flow (L/minute)	40	40	40	40	40	50	40	40	40	40	30	20
RR (breaths/minute)	39	22	26	24	24	31	32	30	30	23	26	20
SpO_2_ (%)	96	94	95	94	95	96	97	97	98	98	99	100
etCO_2_ (mmHg)	44	37	40	42	46	48	46	49	48	45	43	46
V_T_ (mL)				220	210	210	230	220	220	230	230	230
Arterial blood gases												
pH		7.47	7.43	7.41	7.37	7.40	7.38	7.39	7.42	7.42	7.42	7.42
PaCO_2_ (mmHg)		50	53	58	68	66	70	66	61	59	59	60
PaO_2_ (mmHg)		61	73	68	77	80	86	79	91	93	72	85
HCO_3_^−^ (mmol/L)		53.1	34.7	35.9	38.5	40.1	40.1	38.8	39.1	37.9	36.9	37.8

Abbreviations: *etCO*_*2*_ end-tidal carbon dioxide, *F*_*I*_*O*_*2*_ fraction of inspiratory oxygen, *HFT* high-flow oxygen via tracheostomy, *HCO*_*3*_^*−*^ bicarbonate ion, *PaCO*_*2*_ partial pressure of arterial carbon dioxide, *PaO*_*2*_ partial pressure of arterial oxygen, *RR* respiratory rate, *SpO*_*2*_ arterial oxygen saturation by pulse oximetry, *V*_*T*_ tidal volume (measured by Wright respirometer)

We evaluated tracheal pressure during HFT using a flow analyzer (CITREX®, TOKIBO, Co. Ltd, Tokyo, Japan) to measure airway pressure and at the entrance of the tracheostomy tube. The measured values were as follows: 0.21– 0.3 cmH_2_O, 0.21–0.56 cmH_2_O, 0.54–0.91 cmH_2_O, 0.76–2.01 cmH_2_O, 1.17–2.01 cmH_2_O, and 1.76–2.01 cmH_2_O at 10 L/minute, 20 L/minute, 30 L/minute, 40 L/minute, 50 L/minute, and 60 L/minute, respectively. The airway pressure was continuously positive and did not become negative even during inspiration. These results suggest that HFT reduces inspiratory effort. Under respiratory support with HFT and physiotherapy rehabilitation, our patient was successfully weaned from the ventilator. He was discharged from the ICU to the general ward on ICU day 51. His physiotherapy rehabilitation continued for 1 month. He was discharged to home on day 86 of hospitalization and returned for a follow-up visit.

## Discussion

To the best of our knowledge, this report describes the first attempt to administer HFT support during weaning from prolonged mechanical ventilation in patients with restrictive pulmonary dysfunction. Our data suggest that HFT was useful for ventilator weaning. The two patients we weaned from mechanical ventilation exhibited distinctive restriction of the respiratory muscles. HFT increased alveolar ventilation by increasing tidal volume, resulting in reduced respiratory rate and PaCO_2_. Support of inspiration by HFT may also be beneficial for patients who have low tidal volume due to tachypnea or rapid shallow breathing. In our second patient, PaCO_2_ increased from 58 to 72 mmHg after switching from HFT to a T-piece. Then, PaCO_2_ returned to the previous level after switching back to HFT. Even a small reduction of inspiratory effort was expected to cause an increase in the tidal volume as the flow of the HFT was set to the same flow as the ventilator. We think that the main mechanisms of HFT involve reduction of inspiratory effort and increase in tidal volume in patients with restrictive pulmonary dysfunction. As a result, the work of breathing was expected to decrease due to the patient’s slower and deeper breathing. HFT may support inspiration by delivering a high flow and play an important role in decreasing the work of breathing in patients with restrictive pulmonary dysfunction. In a randomized crossover comparison between HFT and T-piece by Corley *et al.* [4], the mean airway pressure during HFT was significantly increased compared with the T-piece 15 minutes after HFT initiation. However, their data revealed no significant difference in end-expiratory lung volume (EELV) between HFT and the T-piece. In contrast to our cases, their patients lacked restrictive pulmonary dysfunction. The absence of dysfunction in these patients might explain the similar EELV values measured with HFT and a T-piece. In our cases, PaCO_2_ and respiratory rate were gradually reduced during HFT, whereas tidal volume gradually increased.

HFNC clears the upper airways of expired air and reduces dead space [5, 6] while concomitantly reducing the work of breathing and PaCO_2_ levels [7]. However, the HFT system results in a more open circuit during inspiration and expiration compared with HFNC. However, this case report demonstrated that HFT increased tidal volume by reducing inspiratory effort in patients with restrictive pulmonary dysfunction.

## Conclusions

The use of HFT was effective for weaning from prolonged mechanical ventilation in patients with restrictive pulmonary dysfunction. HFT may reduce the inspiratory effort and enhance tidal volume by delivering high-flow oxygen and facilitate weaning from ventilators in these patients. We recommend that the first setting of HFT flow is the same as the maximum inspiratory flow of the ventilator during pressure support ventilation, and the duration of HFT is gradually extended in combination with physiotherapy rehabilitation. Further studies are needed to elucidate the efficacy of HFT during weaning from prolonged mechanical ventilation in a larger population of patients with restrictive pulmonary dysfunction.

## Abbreviations

EELV: End-expiratory lung volume
F_I_O_2_: Fraction of inspired oxygen
HCO_3_^−^: Bicarbonate ion
HFNC: High-flow oxygen through nasal cannula
HFT: High-flow oxygen via tracheostomy
ICU: Intensive care unit
PaCO_2_: Partial pressure of arterial carbon dioxide
PaO_2_: Partial pressure of arterial oxygen
PEEP: Positive end-expiratory pressure
